# Relationship between the foot progression angle and eversion and exercise-related lower leg pain

**DOI:** 10.1186/1757-1146-5-S1-O44

**Published:** 2012-04-10

**Authors:** Tine M Willems, Erik Witvrouw, Dirk De Clercq, Philip Roosen

**Affiliations:** 1 Department of Rehabilitation Sciences and Physiotherapy, Ghent University, Ghent, 9000, Belgium; 2 Department of Movement and Sport Sciences, Ghent University, Ghent, 9000, Belgium

## Background

In clinical practice, out-toeing is often linked with an increased eversion and an increased medial pressure distribution. However, in the literature, there is little evidence for this relationship. On the other hand, as an increased eversion and an increased medial pressure distribution have been detected as risk factors for exercise-related lower leg pain (ERLLP) [1,2], and if the foot progression angle is related to these parameters, the occurrence of ERLLP could be due to an increased foot progression angle. The purpose of this study was therefore 1) to investigate the relationship between the foot progression angle and the amount of eversion and the medio-lateral pressure distribution and 2) to check if the foot progression angle is a risk factor for ERLLP.

## Materials and methods

3D gait kinematics combined with plantar pressure profiles were collected from 400 healthy physical education students. After this evaluation, all sports injuries were registered by a sports physician during 3 years. Relationship between foot progression angle and the amount of eversion and the medio-lateral pressure distribution were tested with Pearson correlation. Cox regression analysis was used to investigate the effect of the foot progression angle on the development of ERLLP.

## Results

Forty-six subjects developed ERLLP and 29 of them developed bilateral symptoms thus giving 75 symptomatic lower legs. Bilateral lower legs of 167 subjects who developed no injuries in the lower extremities served as controls. Pearson correlation showed no significant correlation between the foot progression angle and the eversion excursion (*R*=.121) and the medio-lateral pressure distribution (*R*=.116). Cox regression analysis showed no significant differences for the foot progression angle between the injured and uninjured subjects.

## Conclusions

The findings of this study suggest that the foot progression angle is not a risk factor for ERLLP as the foot progression angle is not related to the amount of eversion and the medio-lateral pressure distribution.

## References

[B1] Willems TM De Clercq D Delbaere K A prospective study to gait related risk factors for exercise-related lower leg pain Gait Posture 2006 23 91 98 10.1016/j.gaitpost.2004.12.004 16311200

[B2] Willems TM Witvrouw E De Cock A Gait-related risk factors for exercise-related *lower-leg* pain during shod running Med Sci Sports Exerc 2007 39 330 339 10.1249/01.mss.0000247001.94470.21 17277598

